# Is thrombolysis beneficial in elderly patients with minor ischemic stroke?

**DOI:** 10.3389/fstro.2024.1430261

**Published:** 2024-07-03

**Authors:** Halvor Naess

**Affiliations:** Department of Neurology, Haukeland University Hospital, Bergen, Norway

**Keywords:** cerebral infarction, minor stroke, major stroke, elderly, thrombolysis

## Abstract

**Introduction:**

A pooled analysis of data from randomized controlled trials showed that thrombolysis is an effective treatment in patients older than 80 years of age with acute ischemic stroke. However, the outcomes in daily clinical practice may differ from those observed in randomized controlled trials. Thus, the present study aimed to compare the short-term outcomes of patients older than 80 years of age with ischemic stroke or transient ischemic attacks (TIA) admitted to Haukeland University Hospital in Norway, examining thrombolysis vs. non-thrombolysis treatment in patients.

**Methods:**

All patients with acute ischemic stroke or TIA who were older than 80 years of age and admitted to Haukeland University Hospital within the 4.5-h window after stroke onset between 2006 and 2020 were prospectively included in this observational study. Patients who received thrombolysis were compared to patients who did not receive thrombolysis. The endpoint was a modified Rankin Scale (mRS) score on day 7 or discharge if earlier. The National Institutes of Health Stroke Scale (NIHSS) scores were recorded repeatedly during their hospital stays.

**Results:**

In total, 808 patients were included. Thrombolysis was given to 393 (49%) patients. In patients with an NIHSS score of <3 (minor ischemic stroke) at admission, thrombolysis was associated with worse short-term outcomes (β = 0.13, *p* = 0.03), whereas thrombolysis was associated with better short-term outcomes in patients with an NIHSS score of ≥3 (major ischemic stroke) at admission (β = 0.12, *p* = 0.003). Thrombolysis appeared to be associated with neurological worsening in patients with an NIHSS score of <3 at admission. Excluding patients who underwent a thrombectomy did not change the results.

**Conclusion:**

In elderly patients with major ischemic stroke, thrombolysis was associated with better short-term outcomes. However, in patients with minor ischemic stroke, thrombolysis was associated with worse short-term outcomes. Several reasons for this discrepancy are discussed.

## Introduction

Several studies have shown that thrombolysis is an effective treatment for acute cerebral infarction (National Institute of Neurological Disorders and Stroke rt-PA Stroke Study Group, 1995; Hacke et al., 2008). A pooled analysis of data from randomized controlled trials (RCTs) showed that thrombolysis is also an effective treatment in patients older than 80 years of age (Bluhmki et al., 2020). The study included 1,699 patients from 7 RCTs. However, the outcomes in daily clinical practice may differ from those observed in RCTs. The European Stroke Organisation (ESO) guidelines, as well as the Norwegian guidelines (Helsedirektoratet, 2020), recommend thrombolysis to patients older than 80 years of age with acute cerebral infarction (Berge et al., 2021).

The effect of thrombolysis in patients with minor ischemic stroke is uncertain (Asdaghi et al., 2021). Several studies showed no difference in terms of functional outcome between thrombolysis or antiplatelet treatment in patients with acute minor cerebral infarction (Khatri et al., 2018; Wang et al., 2021; Chen et al., 2023; Monday et al., 2023; Sykora et al., 2023). However, a *post-hoc* analysis of the International Stroke Trial (IST)-3 trial showed significantly better functional outcomes in patients with acute minor cerebral infarction who were treated with thrombolysis (Khatri et al., 2015). In Norway, the frequencies of thrombolysis in patients with acute minor cerebral infarction vary from 10% to 50% between hospitals (Fjaertoft, 2023). The ESO guidelines do not recommend thrombolysis for patients with acute minor cerebral infarction (Berge et al., 2021). The Norwegian guidelines do not address thrombolysis for acute minor cerebral infarction (Helsedirektoratet, 2020). More research is needed to shed light on the different approaches to acute minor cerebral infarction. This is particularly important for elderly patients who are often frail, and the quality of evidence for thrombolysis in frail patients with acute cerebral infarction is very low according to the ESO guidelines (Berge et al., 2021).

The purpose of this study was to compare the short-term outcomes in ischemic stroke patients older than 80 years of age who were admitted within 4.5 h of stroke onset and treated with or without thrombolysis, in relation to their National Institutes of Health Stroke Survey (NIHSS) scores at admission.

## Methods

All consecutive patients with acute cerebral infarction and transient ischemic attacks (TIA) admitted to the Stroke Unit, Department of Neurology, Haukeland University Hospital between February 2006 and December 2020 were prospectively registered in a database (The Bergen NORSTROKE Registry). Cerebral infarction was defined according to the Baltimore–Washington Cooperative Young Stroke Study criteria, including neurological deficits lasting more than 24 h because of ischemic lesions or TIAs, where computed tomography (CT) or magnetic resonance imaging (MRI) showed infarctions related to the clinical findings (Johnson et al., 1995). TIA was defined as neurological deficits lasting <24 h, and no infarction was observed on CT or MRI scans related to the clinical findings.

The present observational study includes all patients older than 80 years of age with either acute cerebral infarction or TIA and who were admitted to Haukeland University Hospital between February 2006 and December 2020within the 4.5-h window of known stroke onset and were eligible for thrombolysis. Stroke mimics are excluded. Patients not treated with thrombolysis received dual antiplatelet therapy (DAPT) in the emergency department. Eligible patients underwent thrombectomy.

Isolated acute cerebral infarctions were defined as lacunar infarctions if <1.5 cm and located subcortical or in the brainstem. All other acute cerebral infarctions were defined as non-lacunar infarctions, which include embolic infarctions and non-lacunar *in situ* thrombosis (Wessels et al., 2006).

The NIHSS was used to assess stroke severity at admission. The NIHSS scores were obtained repeatedly during the first 24 h of admission, day 2, day 3, and day 7 (or on discharge if discharged earlier). Short-term outcomes were determined by the modified Rankin Scale (mRS) score on day 7 or on discharge if discharged earlier.

Neurological worsening during the hospital stay was defined as at least one NIHSS score being 3 points or higher than the NIHSS score on admission during the hospital stay. Another variable registered change in the NIHSS score at admission and the next NIHSS score in order to detect very early neurological worsening.

Prior risk factors and diseases were defined according to a predefined protocol: angina pectoris, myocardial infarction, intermittent claudication, hypertension, diabetes mellitus, and smoking. Current smoking was defined as smoking at least one cigarette per day. Diabetes mellitus was considered present if the patient was on a glucose-lowering diet or on medication. Hypertension, angina pectoris, myocardial infarction, and peripheral artery disease were considered present if diagnosed by a physician any time before stroke onset. Prior medication including taking an anticoagulant was registered. The time from stroke onset to admission was registered.

Etiology was determined by the Trial of ORG 10172 in Acute Stroke Treatment (TOAST) classification and classified as large artery atherosclerosis, cardioembolism, small vessel disease, other, and unknown (Adams et al., 1993).

### Statistics

Locally weighted scatterplot smoothing (LOWESS) curves displaying the short-term outcome (mRS) according to the NIHSS score at admission were obtained. Student's *t*-test and chi-square analyses were performed when appropriate. Stepwise backward linear regression analyses were performed with mRS scores on day 7 or at discharge if earlier as the dependent variable and with the following independent candidate variables: sex, age, NIHSS score at admission, anticoagulation, and time from stroke onset to admission. STATA 14.0 (Statacorp, College Station, Texas, USA) was used for analyses.

## Results

In total, 680 patients with acute ischemic stroke and 128 patients with TIA were included. Of these, 393 (49%) patients underwent thrombolysis.

Table 1 shows demographic data. Patients who received thrombolysis had higher NIHSS scores on admission (*p* < 0.001), higher mRS scores after 1 week (*p* < 0.001), and shorter times from stroke onset to admission (*p* < 0.001). Few patients treated with thrombolysis received anticoagulation before stroke onset (*p* < 0.001). More patients treated with thrombolysis experienced neurological worsening during the hospital stay (*p* = 0.002), but the number of patients with very early neurological worsening was similar in both groups.

**Table 1:** Demographics of ischemic stroke patients >80 years treated with or without thrombolysis.

	**Thrombolysis**		**No thrombolysis**		***p***
Age (mean, *SD*)	86.3	4.3	86.9	4.7	0.07
Female (*N*, %)	178	45	157	38	0.03
Male (*N*, %)	215	55	258	62	
NIHSS on admission (mean, *SD*)	9.5	7.5	5.4	6.9	<0.001
mRS after 1 week (mean, *SD*)	3.2	1.7	2.6	1.8	<0.001
Neurological worsening (*N*, %)	103	26	72	17	0.002
Prior cerebral infarction (*N*, %)	46	12	59	14	0.28
Prior myocardial infarction (*N*, %)	87	22	84	20	0.51
Hypertension (*N*, %)	236	60	270	65	0.14
Diabetes mellitus (*N*, %)	64	16	59	14	0.43
Atrial fibrillation (*N*, %)	172	44	205	49	0.11
Smoking (*N*, %)	30	8	33	9	0.79
Anticoagulation (*N*, %)	21	5	84	20	<0.001
Time from ictus to door (mean, *SD*)	91	55	106	60	<0.001
Thrombectomy (*N*, %)	70	18	28	7	<0.001
TOAST (*N*, %)					0.15
Atherosclerosis	25	6	37	9	
Cardiac embolism	168	43	191	46	
Small vessel disease	19	5	24	6	
Other	0	0	2	0.5	
Unknown	178	46	159	39	

NIHSS, National Institutes of Health Stroke Scale; mRS, modified Rankin Scale; TOAST, Trial of ORG 10172 in Acute Stroke Treatment.

**Figure 1:**
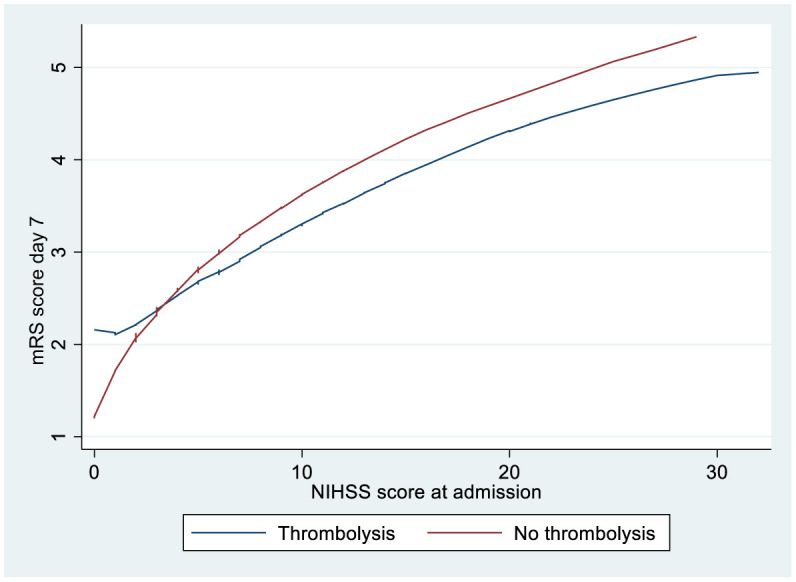
LOWESS smoother curves showing mRS score according to the NIHSS score at admission for ischemic stroke patients >80 years treated with or without thrombolysis. LOWESS, locally weighted scatterplot smoothing; mRS, modified Rankin Scale; NIHSS, National Institute of Health Stroke Scale.

Figure 1 shows that patients with an NIHSS scores of >3 on admission had lower mRS scores after 1 week when treated with thrombolysis. Linear regression analysis shows that patients receiving thrombolysis did significantly better when adjusting for the NIHSS score on admission and other confounders if the NIHSS score was ≥3 (Table 2).

**Table 2:** Linear regression with mRS as a dependent variable if the NIHSS score was <3 at admission.

	**β**	***p***
Age	0.30	<0.001
Male	0.15	0.008
NIHSS	0.22	<0.001
Thrombolysis	0.13	0.03

mRS, modified Rankin Scale; NIHSS, National Institute of Heath Stroke Scale.

Figure 1 shows that patients with an NIHSS score of <3 on admission had higher mRS scores after 1 week when treated with thrombolysis. Linear regression analysis shows that patients receiving thrombolysis did significantly worse when adjusting for the NIHSS score on admission and other confounders if the NIHSS score was <3 (Table 3).

**Table 3:** Linear regression with mRS on discharge as a dependent variable if the NIHSS score was ≥3 on admission.

	**β**	***p***
Age	0.19	<0.001
Male	−0.01	0.7
NIHSS	0.45	<0.001
Thrombolysis	−0.12	0.003

mRS, modified Rankin Scale; NIHSS, National Institute of Heath Stroke Scale.

**Figure 2:**
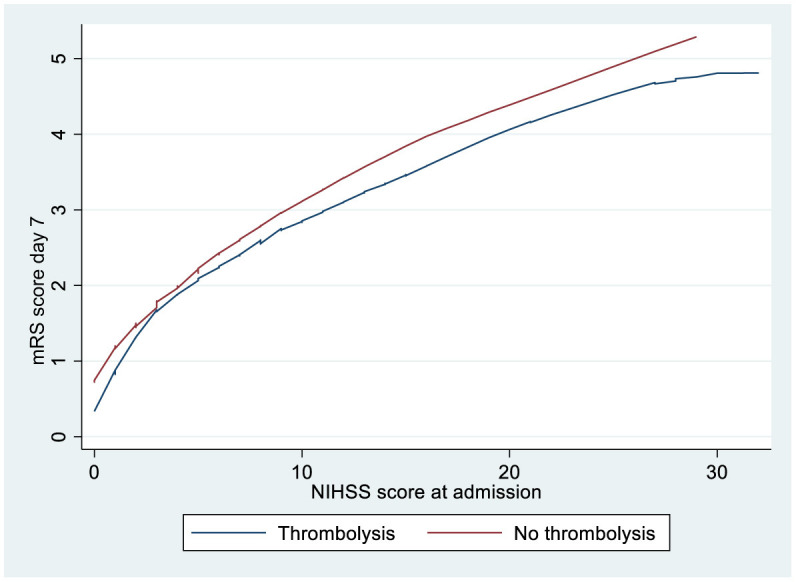
LOWESS smoother curves showing mRS score according to the NIHSS score at admission for ischemic stroke patients >80 years treated with or without thrombolysis and no neurological worsening. LOWESS, locally weighted scatterplot smoothing; mRS, modified Rankin Scale; NIHSS, National Institute of Health Stroke Scale.

Figure 2 shows that patients without neurological worsening had lower mRS scores after 1 week if treated with thrombolysis irrespective of the NIHSS score at admission.

Patients with no hemorrhagic transformation, who did not undergo thrombectomy, and with lacunar or non-lacunar stroke who were investigated separately did not change the inverse outcome relation between patients treated with or without thrombolysis according to their NIHSS score at admission.

## Discussion

Among elderly patients with cerebral infarction and an NIHSS score of ≥3, patients treated with thrombolysis had better short-term outcomes than patients not treated with thrombolysis. This is in line with a pooled analysis of data from randomized controlled trials, which showed that thrombolysis is an effective treatment in patients older than 80 years. However, the benefit was not statistically significant for minor cerebral infarction in that pooled analysis (Bluhmki et al., 2020).

The effect of thrombolysis in patients with minor cerebral infarction is uncertain irrespective of age (Asdaghi et al., 2021). One possible cause for this uncertainty is that randomized controlled trials included relatively few patients with minor cerebral infarction. Another possible cause is that the mRS score, which is often used as the primary endpoint, is a course scale for minor functional impairments.

Recently, several RCTs and observational studies have addressed the effect of thrombolysis in acute minor cerebral infarction irrespective of age. One RCT found that DAPT was not inferior to thrombolysis in patients with an NIHSS score of ≤ 5 at admission (Chen et al., 2023). A meta-analysis did not reveal any difference between thrombolysis or DAPT in acute minor cerebral infarction related to functional outcomes (Monday et al., 2023). Moreover, an observational study did not find any difference between thrombolysis and antiplatelet treatment in patients with acute minor cerebral infarction regarding functional outcomes (Wang et al., 2021). One observational study found no difference between patients receiving thrombolysis and those not receiving thrombolysis with an NIHSS score of ≤ 3 at admission, but functional outcomes were significantly better in patients treated with thrombolysis with an NIHSS score of 4–5 at admission (Lei et al., 2024).

We found that thrombolysis was associated with worse short-term outcomes in patients with minor cerebral infarction (an NIHSS score of <3 on admission) after adjusting for the NIHSS score on admission, whereas the opposite was the case for patients with the NIHSS score of ≥3 at admission as stated earlier. Similar contrasting outcomes between minor and major ischemic stroke appeared to be the case when lacunar infarction, non-lacunar infarction, and the exclusion of intracranial hemorrhagic complications were analyzed separately based on LOWESS analyses among our patients.

However, excluding patients who experienced neurological worsening after admission showed that thrombolysis appeared to be associated with better outcomes irrespective of the NIHSS score at admission based on LOWESS analysis. Thus, our finding that thrombolysis leads to worse outcomes in patients with minor cerebral infarction is driven by patients experiencing neurological worsening after admission. Interestingly, an RCT study comparing alteplase with DAPT in acute minor cerebral infarction showed that early neurological deterioration was significantly more common in the thrombolysis group (Chen et al., 2023) and an observational study found that compared to DAPT, thrombolysis was significantly associated with early deterioration in patients with non-cardioembolic stroke and the NIHSS score of ≤ 3 at admission (Sykora et al., 2023).

There is no consensus regarding the definition of acute minor cerebral infarction. Several studies define minor stroke as an NIHSS score of ≤ 5 at admission. However, our findings and the findings in an observational study (Sykora et al., 2023) suggest that an NIHSS score of ≤ 3 at admission may be a better definition because thrombolysis is possibly detrimental in patients with an NIHSS score of ≤ 3 at admission but beneficial in patients with an NIHSS score of ≥4 at admission.

There are several possible explanations for our findings. Perhaps some patients received thrombolysis because they deteriorated within the time window for thrombolysis. However, there was no difference in the number of patients with very early neurological worsening whether thrombolysis was administered or not.

It is well known that thrombolytic therapy may cause paradoxical activation of thrombin, leading to clot formation (McCartney et al., 2019). This is possibly a bigger problem with a minor ischemic cerebral infarction than a major cerebral infarction. It is conceivable that many patients with minor cerebral infarction have already experienced spontaneous clot lysis and that thrombolysis therefore only has a potential negative effect through paradoxical clot formation.

Another reason for a worse prognosis when thrombolysis is given to patients with minor cerebral infarction is that alteplase can be neurotoxic (Harston et al., 2010). However, this is controversial as alteplase does not increase brain injury after mechanical middle cerebral artery occlusion in the rat (Sutherland and Buchan, 2013).

The authors of an RCT study suggested that early deterioration in the thrombolysis group compared to the DAPT group could be due to thrombus progression. Alteplase has a short half-life, whereas DAPT provides a continuous antiplatelet effect and may prevent recurrent stroke (Chen et al., 2023).

The strengths of this study include the many patients treated and monitored according to the same protocol for many years. A major limitation is that this is an observational study. Thus, unknown biases may have influenced the choice of treatment with or without thrombolysis. The monitoring of patients may also have been influenced by the choice of treatment, although equal monitoring was mandated according to our guidelines. Another limitation is that we did not record why patients did not receive thrombolysis. Recent studies show that thrombolysis may be beneficial in an extended window of up to 9 h after stroke onset. We do not have enough data in our database to investigate thrombolysis in the extended time window.

In conclusion, in elderly patients with major cerebral infarction, thrombolysis was associated with better short-term outcomes. However, in patients with minor ischemic stroke, thrombolysis was associated with worse short-term outcomes. Several reasons may explain this discrepancy and should be evaluated in future randomized controlled trials.

## Data availability statement

The data analyzed in this study is subject to the following licenses/restrictions: Data can be obtained from the author. Requests to access these datasets should be directed to HN, haln@ihelse.net.

## Ethics statement

The studies involving humans were approved by Regional komité for forskningsetikk, Vest Norge (REK Vest). The studies were conducted in accordance with the local legislation and institutional requirements. Written informed consent for participation was not required from the participants or the participants' legal guardians/next of kin in accordance with the national legislation and institutional requirements.

## Author contributions

HN: Formal analysis, Methodology, Validation, Writing – original draft, Writing – review & editing.
